# An Interoperable System toward Cardiac Risk Stratification from ECG Monitoring

**DOI:** 10.3390/ijerph15030428

**Published:** 2018-03-01

**Authors:** Cristina Soguero-Ruiz, Inmaculada Mora-Jiménez, Javier Ramos-López, Teresa Quintanilla Fernández, Antonio García-García, Daniel Díez-Mazuela, Arcadi García-Alberola, José Luis Rojo-Álvarez

**Affiliations:** 1 Department of Signal Theory and Communications and Telematics Systems and Computing, Rey Juan Carlos University, 28942 Madrid, Spain; inmaculada.mora@urjc.es (I.M.-J.); javier.ramos@urjc.es (J.R.-L.); joseluis.rojo@urjc.es (J.L.R.-Á.); 2 Department of Signal Theory and Communications, Carlos III University, 28912 Madrid, Spain; mteresa.quintanilla@gmail.com; 3 Niño Jesús Hospital, 28009 Madrid, Spain; antonio.garcia.garcia@salud.madrid.org; 4 Doce de Octubre Hospital, 28041 Madrid, Spain; daniel.diez@salud.madrid.org; 5 Unit of Arrhythmias, University Hospital Virgen de la Arrixaca, 30120 Murcia, Spain; arcadi@secardiologia.es; 6 Center for Computational Simulation, Universidad Politécnica de Madrid, Boadilla, 28223 Madrid, Spain

**Keywords:** electronic health records, archetypes, semantic interoperability, heart rate turbulence, heart rate variability, cardiovascular risk stratification, web system

## Abstract

Many indices have been proposed for cardiovascular risk stratification from electrocardiogram signal processing, still with limited use in clinical practice. We created a system integrating the clinical definition of cardiac risk subdomains from ECGs and the use of diverse signal processing techniques. Three subdomains were defined from the joint analysis of the technical and clinical viewpoints. One subdomain was devoted to demographic and clinical data. The other two subdomains were intended to obtain widely defined risk indices from ECG monitoring: a simple-domain (heart rate turbulence (HRT)), and a complex-domain (heart rate variability (HRV)). Data provided by the three subdomains allowed for the generation of alerts with different intensity and nature, as well as for the grouping and scrutinization of patients according to the established processing and risk-thresholding criteria. The implemented system was tested by connecting data from real-world in-hospital electronic health records and ECG monitoring by considering standards for syntactic (HL7 messages) and semantic interoperability (archetypes based on CEN/ISO EN13606 and SNOMED-CT). The system was able to provide risk indices and to generate alerts in the health records to support decision-making. Overall, the system allows for the agile interaction of research and clinical practice in the Holter-ECG-based cardiac risk domain.

## 1. Introduction

Sudden cardiac death (SCD) can be defined as a natural death with cardiac origin. It is given this denomination when no prior fatal condition has been observed, and either (a) it takes place in less than one hour from the observation of the first detected symptoms, or (b) it happens in the next 24 h of having seen the person alive [1,2,3]. SCD is today a major cause of mortality worldwide [1]. In order to reduce its incidence, a number of methods for risk stratification have been proposed. Whereas most SCD episodes are given in patients with previous cardiac disease, they can also occur in people without previous symptoms, and there is no accurate enough method to date to effectively predict SCD in these conditions. The electrocardiogram (ECG) provides us with a number of risk predictors for SCD, but despite the intense research in this field, few of these indices are nowadays used in the clinical routine, as pointed with detail in [4].

On the other hand, clinical decision support systems (CDSSs) have been widely studied in recent decades [5,6,7,8]. In this sense, the patient health information registered in the Electronic Health Records (EHR) can be very useful for healthcare providers in their decision-making processes, due to the large amount of available clinical information, and in sharing data with heterogeneous clinical systems [9].

However, there is a separate evolution among the Electronic Health Record (EHR) and the CDSS due to the lack of maintainability and interoperability between CDSS components in EHRs, and the lack of EHR semantics that capture the highly structured patient data needed in computerized guideline execution and CDSSs [10]. This means that challenges in semantic interoperability and technical infrastructure are still needed [11,12,13]—for instance, the integration and standardization of the formats among different sources, and the formal definition of shared clinical information models together with their terminological binding. To overcome these and other related challenges, the use of clinical terminologies and EHR standards is a strong need, clinical terminologies, such as SNOMED-CT, provide a shared vocabulary to exchange clinical data with full meaning and independently of language differences. On the other hand, the use of EHR standards, such as openEHR or CEN/ISO EN13606, yields a common representation of clinical data. Both terminologies and EHR standards are merged through a process called terminology binding among clinical terms and clinical information models, hence providing the standardized information with semantics [13]. Apart from that, HL7 provides a framework for the syntactic exchange of electronic health information that supports clinical practice [14]. Previous studies have focused on health care and risk management systems that generate alert messages based on the standards of, for example, SNOMED-CT and HL7 [15].

Our long-term objective is to create an advanced intelligent and interoperable system for supporting the cardiac risk stratification from ECG monitoring and EHR data for SDC, capable of integrating both research and clinical practice from multiple hospitals collaborating in this domain. This is a complex task, which requires advances in quite different fields, including ECG-advanced signal processing, the design of centralized intelligent engines, interoperability considerations, and precise medical descriptions for this wide, complex, and intensely studied domain [4].

In this work, we generated the technical and clinical domain descriptors, together with the interoperability elements, necessary to create a system in which a cardiovascular risk model can grow with clinical practice. For this purpose, three subdomains have been created. First, a patient data summary that represents the data that can be extracted from the EHR or even from the Hospital Information System (HIS), and that can be potentially useful for risk stratification, is included. Second, a low-complexity subdomain is given based on the heart rate turbulence (HRT), and provides us with a reduced set of risk indices derived from the ECG in Holter recordings from simple signal processing and with simple risk rules defined in the literature [16]. Third, a high-complexity subdomain is given based on the heart rate variability (HRV) [2], as a large number of indices have been proposed, and no clear guidelines have been established to date. Therefore, focusing on both HRT and HRV subdomains enables us to scrutinize the main interoperability issues as adequate archetypes, standards, and clinical terminologies.

**Figure 1:**
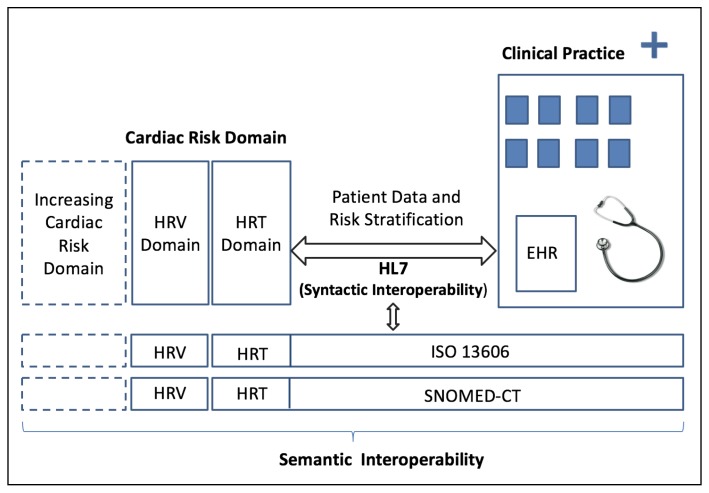
Functional schema of the proposed sudden cardiac death (SCD) risk stratification system.

The proposed SCD Risk Stratification System based on the HRT and HRV indices from ECG monitoring for Clinical Decision Support (CDS) can be used by different EHRs, thanks to the use of SNOMED-CT, CEN/ISO EN13606, and HL7 standards. Our system (1) supports the use of clinical standards for CDS, (2) supports the scientific assessment and improvement of HRT and HRV knowledge within their domains by developing a structured database, paving the way toward a progressive domain expansion, and (3) provides technological support for implementing SCD risk stratification in clinical practice (see Figure 1 for a schematic view of the proposed SCD system). Therefore, the system provides a framework where the interoperability layers and the description of the cardiovascular risk model grow together and are in line with clinical practice. In order to analyze the performance, scope, and limitations of the proposed system, we analyzed the data from a clinical trial of SCD risk prediction conveying patient data, HRT, and HRV measurements in patients of University Hospital Virgen de la Arrixaca (UHVA) in Murcia, Spain. We also tested the system interoperability with these data in an EHR from a different medical institution, namely, University Hospital 12 de Octubre (H12O) in Madrid, Spain.

Section 2 presents a brief background on ECG indices for SCD risk stratification and summarizes the background on clinical standards used in this work. Section 3 describes the fundamentals of the proposed system and its elements and presents the database description and in-hospital system validation test. Discussion and conclusions are finally stated in Section 4 and Section 5.

## 2. Materials and Methods

### 2.1. ECG Indices for SCD Risk Stratification

Current SCD risk stratification techniques can be divided into three groups [4]: (a) invasive techniques, which include tests during electrophysiological study using catheters; (b) noninvasive techniques, such as medical images, ECG with 12 lead recordings, Holter ECG, several baroreflex tests, and high-resolution ECG; and (c) a variety of additional tests, including genotype or stress, which can be useful in patients with specific cardiopathies [17,18]. The left ventricular ejection fraction (LVEF) is the most commonly used index to identify patients with high-risk of SCD, but other noninvasive techniques and measurements based on ECG Holter monitoring have been proposed in the literature. This diagnostic method records ECG signals from 24 to 48 h in two or three chest leads. Relevant electrophysiological events can be detected in Holter recordings, including a number of advanced markers for SCD risk stratification, which alterations can promote ventricular arrhythmias. Probably the mostly studied examples of these ECG markers are late potentials, heart rate variability (HRV), T–wave alternans (TWAs), deceleration capacity, and HRT [4].

There is no consensus about the best convenience on signal processing algorithms and their adjustment in SCD risk stratification from ECG monitoring [19]. Hereinafter, we will use the term “cardiac risk stratification” (CRS) to refer to SCD risk stratification. The computational techniques used for yielding SCD markers from the ECG are extremely diverse, and they should be taken into consideration for any domain description on SCD. Moreover, the scientific evidence, as given by clinical population studies in representative patient databases, has not always been solid enough to support the different existing algorithms. Additionally, the limitations of many of these clinical studies have not allowed for their reliable generalization [4]. For all the above reasons, in the present work, we focus on subdomain descriptions of HRT and HRV as risk indices for SCD.

HRT has been pointed out as a promising, strong, and noninvasive index CRS after acute myocardial infarction [16]. The term HRT describes the phenomenon of short-term fluctuation in the heart cycle during beats after a premature ventricular complex (PVC). The HRT is usually assessed based on ECG monitoring for 24 h (24 h Holter recordings) in which the PVCs are identified. Then, a 20-beat tachogram (representation of the beat intervals as a time series) per PVC is built, and two indices per tachogram are measured. The turbulence onset (TO) index quantifies the sinus acceleration after a PVC, and the turbulence slope (TS) index measures the rate of sinus deceleration following the sinus acceleration [16]. In most clinical studies, CRS using HRT indices is inferred using just TS and TO. According to guidelines [16], we considered the cut-off values of 0% and 2.5 ms/RR-intervals for TO and TS, respectively, to classify HRT indices into three categories: Category 0 is obtained when both TO and TS are normal (i.e., TO < 0 and TS > 2.5); Category 1 is obtained when TO > 0 or TS < 2.5, i.e., either TO or TS is abnormal, and Category 2 is obtained when both indices are abnormal, i.e., TO > 0 and TS < 2.5.

HRV measures the temporal dispersion of series of consecutive beats. The short-term and the long-term HRV are associated with the heart rate control by the autonomic nervous system and with the complex auto-regulation cardiovascular mechanisms, respectively [20]. Many other different systems and organs (including heart, digestive system, kidney, or respiratory system) also contribute to the modulation of the heart rate through a complex dynamic equilibrium with the cardiovascular system mechanisms [21,22,23]. A really great number of indices have been proposed to quantify and measure the HRV. The interested reader can see [4,20] for reviews. As a summary for this work, we used the conventional grouping for HRV indices, which are usually considered as statistical indices (obtained by simple statistical measurements in the beat time series or its derivative), geometrical indices (obtained from graphical interpretations related to the density function or its transformations), and spectral indices (obtained from power measurements in spectral transformations of the beat series).

### 2.2. Clinical Interoperability Standards

According to the Institute of Electrical and Electronics Engineers (IEEE), interoperability is defined as the capacity of several systems or elements to share and use information. Two types of interoperability can be defined, namely, syntactic [24] and semantic interoperability [25]. The first one refers to the capacity of communicating and exchanging data among two or more systems. Toward that end, specified data formats and communication protocols such as XML or SQL are required. In this work, we use the HL7 standard. On the other hand, semantic interoperability is the ability of a system to automatically understand the exchanged information with meaning and accuracy regardless of the end users. It is still a major challenge, specially in public health systems [26]. The CEN/ISO EN13606 standard is used here to achieve semantic interoperability. We next summarize the main concepts of the standards used in this work to achieve both syntactic and semantic interoperability, namely, HL7, CEN/ISO EN13606, and SNOMED-CT.

The HL7 standard provides a comprehensive framework for the sharing of electronic health information supporting clinical practice [24]. It allows for the transmission of clinical and administrative information based on a common format message [27]. An HL7 message is the minimum unit of data exchange, which follows a hierarchical structure. Specifically, the message is comprised of a sequence of segments, using three characters to identify the message type. The semantic content of a message is described and transmitted by elements defined in segments. For example, the admission, discharge, and transfer (ADT) code is used to transmit administrative data. The following segments may appear in an ADT message: MSH (message header), EVN (even type), PID (patient ID), and PV1 (patient visit) [24]. The structure of more HL7 messages can be checked in [24].

The CEN/ISO EN13606 Standard aims to achieve the semantic interoperability by using a dual model architecture. The main advantage of the dual model architecture. The main advantage of the dual model is that knowledge (archetypes) is upgraded when it changes, whereas the reference model (information) remains unaltered [25]. A reference model is an object-oriented model that comprises a small set of classes defining the generic building blocks to construct EHRs. It is used to represent the generic and stable properties of health record information [25]. Archetypes are formal definitions of clinical concepts in the form of structured and constrained combinations of the entities of a reference model, providing a semantic meaning to a reference model structure [25,28,29]. They represent a specific clinical concept, such as blood pressure measurement.

The most relevant international resource repository is the openEHR Clinical Knowledge Manager (CKM), promoted by the openEHR Foundation (http://openehr.org/ckm/). The collection contains a set of reviewed and validated archetypes, and is constantly growing. This repository has 513 active archetypes in August 2017, which are in various stages of development (draft, revised, and published), and includes clinical and administrative archetypes developed by a large number of clinical and computer experts at an international level.

SNOMED-CT is an standardized and multilingual vocabulary of clinical terminology, resulting from the merging of SNOMED Reference Terminology (SNOMED RT) and Clinical Terms Version 3 [30]. SNOMED-CT probably represents the most complete classification for clinical use, being the reference to terminologies for different health professionals. It consists of a structured collection of health care terms, which are attached to concept codes with multiple definitions per code (for more details, see e.g., [30]).

Our research group has been working for the last several years on the standardization of the cardiac domain from the EHR to provide CRS. Given that this is an extremely wide knowledge domain, our focus is on the indices measuring repolarization heterogeneity and autonomous nervous system control from long-term ECG signal processing. To this aim, we first built an ontology based on the conceptual model of SNOMED-CT for CRS using ECG-derived parameters in the HRT domain [31]. Two practical studies were proposed therein: (a) the practical development of a clinical template based on the HRT ontology, which was implemented in the EHR of University Hospital of Fuenlabrada (Madrid, Spain); (b) a simple application example of clinical decision support involving the EHR and signal processing techniques.

We found two main drawbacks in these practical implementations. First, the HRT template based on the ontology did not achieve semantic interoperability in the EHR, because it was created with no clinical standard to model the clinical domain. Second, the implementation of the HRT form in different clinical systems was very difficult to reach, since changes in commercial systems require political consensus. For the former, a CRS archetype was subsequently built, following the CEN/ISO EN13606 standard, to achieve the interoperability among heterogeneous clinical systems [32]. A simple and helpful way of binding archetypes to the ontology was also proposed [32].

For the latter, we propose in this work an interoperable web-based CDSS that uses archetypes and clinical terminologies. We used the standard HL7 v.2.6 to achieve syntactic interoperability, and CEN/ISO EN13606 to achieve semantic interoperability.

## 3. The Proposed Interoperable System and Results

In this section, we describe the implementation of a risk system based on ECG monitoring (RSEM) for making the interoperability (collection and exchange) of EHR, HRT, and HRV data provided by different health entities. The recommendation in [33] suggests that semantic interoperability is an essential factor for the EHR to improve the quality and safety of patient care, public health, clinical research, and health service management. Interoperability is also necessary to our final goal: to collect a large number of cases under a common framework (structured database) to be able to scientifically assess and improve the knowledge of the CRS, hence allowing for wider and subsequent domain expansions with both clinical practice and research. Note that the availability of a database of this nature will allow for the advantageous use of data analytics for this kind of CDS-based system. We first present the clinical background and the system specifications, which give rise to the system’s functional scheme. The three subdomains definitions for CRS are then described.

### 3.1. System Specifications and Functional Scheme

Our system integrates several parameters and measurements from EHR and Holter, all of which are potential indices of SCD, hence allowing for the creation of increasingly improved risk indices. The process is the following: first, a set of non-Holter variables with prognostic interest are extracted from the EHR (general factor such as age and gender, LVEF, diagnosis and drugs); second, the Holter recording is analyzed and additional indices are automatically obtained (here, related to HRT and HRV); third, a risk scale is built for all of them.

It is important to note the paradoxical state of art of risk stratification from Holter measurements [4]. On the one hand, an always-growing literature exists, supporting scientific evidence on their linkage to risk stratification. On the other hand, multicentric studies trying to make this evidence definitive often fail, mostly due to the complexity of the domain, to the sensitivity to the signal processing, and to the dependence with the stratification data model. This makes risk stratification from Holter to often be left apart on cardiac patients in clinical practice. In this current scenario, the proposed system takes benefit from existing technology to support the CRS research from clinical practice. The daily use of Holter on a wide population, with a homogeneous definition of signal processing, combined with other patient variables standardized in the EHR, will provide risk stratification support. Hence, homogenization and standardization will overcome the limitations of classical risk studies (limited to a few hundred patients), while costs will be reduced and several thousand patients will be reached.

According to previous considerations, the specifications for this work are the following:

1. to create a system, external to the EHR and HIS, that is interoperable with them, thanks to its design following clinical standards;
2. to cover a significant part of the CRS domain from ECG-derived indices measured in Holter recordings, namely, patient summary data, HRT, and HRV subdomains;
3. to be able to grow by including other cardiac risk subdomains;
4. to allow us to perform signal processing;
5. to generate alerts related to the CRS domain, with different levels, and to feed them back into the EHR.

One of the long-term requirements of the RSEM is to model the CRS domain and to describe all the related concepts and data logged on the EHR. For this purpose, an advantageous approach can be given by establishing the required standards starting from the patient data, HRT, and HRV subdomains. This way, if we define the interoperability requirements for these subdomains, these requirements will be readily extended to other SCD risk subdomains, so that other hospitals can integrate and share the existing subdomains descriptions, as well as their use and integration in clinical practice. Since clinical and demographics variables can be also identified as risk factors related to CSR, they are included in our RSEM system.

The availability of the clinical data in the EHR for an SCD risk stratification system is naturally provided by the semantic and the syntactic interoperability. Specifically and described previously, in this work, we considered (1) the standard HL7 for communication purposes; (2) the standard CEN/ISO EN13606 for the archetypes construction; and (3) SNOMED-CT for binding archetypes and the ontology and thus obtaining semantic interoperability.

To achieve the semantic standardization of clinical data from the EHR for providing with CRS, clinician expertise was required. Given that CRS is an extremely wide knowledge domain, our focus was on those indices measuring repolarization heterogeneity and autonomous nervous system control form long-term ECG signal processing. To this aim, clinicians suggested using ECG-derived indices on the HRT domain as a first approximation due to its concise guidelines. This simple subdomain was then completed with a more complex subdomain of SCD, given by HRV indices. Toward that end, several archetypes were developed.

Apart from the construction of a clinical structured database, interoperability among different healthcare systems is also pursued by exporting clinical data as extracts in xml format [32]. These extracts have the same structure and constraints as the built archetypes, so they also provide semantic interoperability.

### 3.2. Subdomain Definitions for CRS

**Figure 2:**
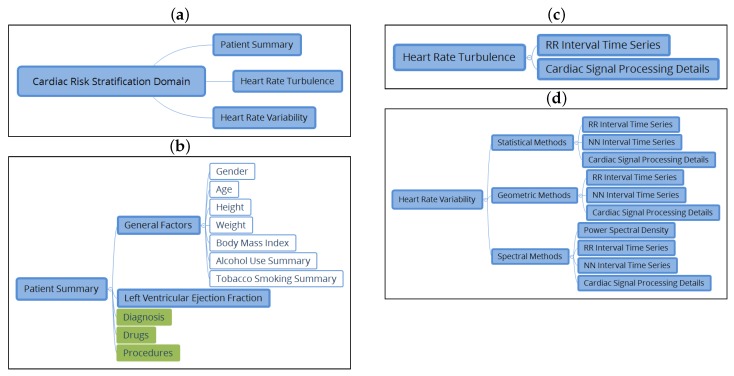
Archetypes used for defining the cardiac risk stratification (CRS) domain (**a**), the patient data subdomain (**b**), and the heart rate turbulence (HRT) (**c**) and heart rate variability (HRV) (**d**) subdomains. Archetypes selected from the Clinical Knowledge Manager (CKM) and used with no modifications are in white, specialized archetypes from the CKM are in green, and archetypes proposed in this work are in blue.

Clinicians in our group defined the subdomain elements (nodes and constraints) needed for achieving a good knowledge representation from a clinical viewpoint. Main archetypes for defining CRS domain are shown in Figure 2a. Each subdomain is next described:

(1) The Patient Summary Subdomain. The description of the patient summary subdomain is shown in Figure 2b, and it is defined by different archetypes. The white color is used to identify those archetypes selected from the CKM and used without making any modifications: gender, age, height, body mass index (BMI), alcohol use summary, and tobacco summary. They were included in a common archetype called *general factors*. The green color identifies the archetypes from the CKM, which requires specialization in order to be used in the CRS domain. The requirements were established with the clinical expert (author AGA). The archetype that models the information of the diagnosis was specialized to reflect the set of pathologies that can influence the HRT or the HRV. Fifteen pathologies were identified: asthma, cerebral damage, depression, diabetes mellitus, myocardial dysfunction, chronic kidney disease, chronic obstructive pulmonary disease, hypothyroidism, myocardial infarction, heart failure, stroke, peripheral neuropathology, Parkinson’s syndrome, overweight, and tetraplegia. The drug archetype also needed to be specialized only to consider drug families by pharmacological activity. A total of 44 families were identified as relevant to the CRS domain. The archetype selected for modeling the information about *procedures* was also specialized. We considered 5 procedures as relevant: cardiac transplantation, coronary revascularization surgery, cardiac resynchronization, pacemaker implantation, and implantable cardioverter defibrillator implantation. Finally, the blue color is used to identify new archetypes: LVEF and patient summary, which compiles with all of the described archetypes related to demographic and clinical information.

(2) The HRT Subdomain. It compiles the information of 24 h Holter recordings in order to infer the CRS in terms of HRT indices, and TS and TO values. The inferred category was also considered as an element in the HRT archetype. We defined and built two more archetypes to model this subdomain (see Figure 2c for details). First, the archetype for the RR interval time series series includes RR record information (the file in common multimedia format for recording equipment), RR series duration (hours, minutes, and seconds), the number of abnormal beats, a field of free text for comments, and the possibility to extend the archetype with other archetypes that complete the information of the previous elements. Second, the archetype of cardiac signal processing details structures information related to the indices of the algorithm used to process the cardiac signal. A free text variable is included to indicate the name of the processed signal, the sampling frequency (Hz), the processing method, interpolation type selection (linear, parabolic, triangular, or text free), and the possibility of extending the archetype with other archetypes that complete the information of the previous elements.

(3) The HRV Subdomain. Because of its complexity, the HRV subdomain has not been previously standardized in the literature. Toward that end, we first describe the archetypes related to HRV measures according to [20] (see Figure 2d for details):

- An archetype of *statistical measures* was created, which includes the value in milliseconds of the SDNN, the SDNN index, and the SDANN indices, calculated from the series of NN intervals, the values of indices NN50, SDS, and RMSS in milliseconds, and pNN50 described as a proportion.
- An archetype of *geometric measurements* that includes the value of the TINN, the triangular index, the differential index, the logarithmic index, and the poincare SD1 and SD2 indices was created.
- An archetype of *spectral measurements* of HRV was created. For the case of short-term analysis, it includes the value of total power, VLF power, LF power, HF power (power values in ${\mathrm{ms}}^{2}$), normalized LF power, normalized HF power, and LF/HF—in the case of an ambulatory analysis, total power, ULF power, VLF power, LF power, HF power, and alpha index.

The HRV subdomain also collects information about the protocol to carry out the measurement: a selection variable to indicate whether the analysis is with ambulatory registration or is short-term, slots to include other archetypes related to the RR and signal processing, and information about the device. The archetype NN interval time series includes information from the NN series used to perform index measurement: the NN signal register (the file in a common multimedia format for the recording equipment), duration (hours, minutes, and seconds), the number of NN intervals, a free text field for comments, and the possibility of extending the archetype with other archetypes that complete the information of previous elements. The archetype power spectral density for the calculation of the power spectral density is used only with the spectral methods to collect information on the method used to calculate the power spectral density. It facilitates with a slot the power to embody the archetype of cardiac signal processing.

The definition of these subdomains, together with a basic implementation, are the key contributions of the present work, as they have been raised from the interaction of Holter signal processing experts and clinical experts, following the guidelines when present or their surrogates otherwise [16,34].

### 3.3. Database Description

Data were assembled from a clinical trial performed in UHVA. The ongoing study aims to evaluate the CRS of a patient population hospitalized for congestive heart failure. A set of variables were taken for each patient, namely, demographic and clinical variables at hospital release, ECG, and echocardiographic variables, and Holter analysis including HRT and HRV measurements.

Table 1 shows the demographic data and cardiac measurements from 24 patients in this study: (1) general factors (age, gender, height, weight, BMI, and tobacco); (2) LVEF; (3) diagnosis (diabetes mellitus, hypertension, hypercholesterol, myocardial infarction); and (4) drugs (β-blocker).

Table 2 shows the inferred risk category based on normal values of certain HRT and HRV indices considered important by clinicians. Measurements of HRT for patients who do not have at least 3 or 5 sinus intervals before the PVC have been excluded [35]. HRV indices have been provided for patients who have segments with more than 90% sinus beats [20]. Evaluation of the remaining patients was not possible.

Normal values of these indices were obtained from [16] for HRT and from [2] for HRV. Bold numbers are used to identify no normal values. For HRT, due to its concise guidelines, the risk evaluation was provided by computing the category using TS and TO values, yielding 3 patients in Category 0, 4 patients in Category 1, and 3 patients in Category 2. The variety of circumstances that can affect HRV is large; hence, in this work, we implemented a simple rule requiring a proportion of indices with abnormally reduced values to define the HRV risk evaluation. Note that this represents a simple surrogate criterion that can be substituted by a more sophisticated and validated risk calculation method. It is important to remark too that an isolated index can often be affected by causes unrelated to SCD (e.g., if the patient exercises more, a higher SDANN value can be obtained). Taking this into account, we propose three categories based on the number of non-normal HRV indices: Category 0 when less than 33% of the indices have non-normal values, Category 1 when 33–66% have non-normal values, and Category 2 when more than 66% have non-normal values.

### 3.4. System Validation

**Figure 3:**
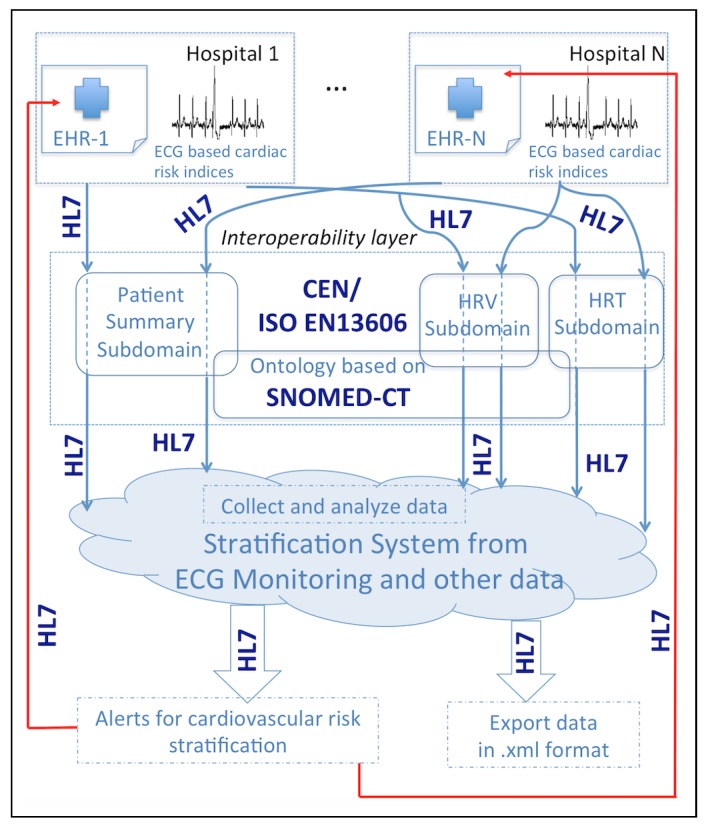
Interoperability schema of the data collection and transmission, as well as the elements and standards involved in the proposed system.

Data from patient summary and EHR were connected with RSEM by using HL7, archetypes, and SNOMED-CT, as described in Section 2.2, see Figure 3 for details. To design RSEM, we use apache, PHP, MySQL, and phpMyAdmin under the web development environment WampServer. Bootstrap and the WampServer development environment with all their functionalities were considered; phpMyAdmin was used to create a MySQL database in a simple way, PHP allowed users to be turned around with Bootstrap to communicate them with the database and to calculate the HRT and HRV evaluation. Everything was housed in an Apache local server.

The RSEM provided with (1) SCD risk stratification based on HRT and HRV indices and (2) alerts appearing in the EHR to support decision-making. The RSEM was able to exchange in real time information with full meaning between an actual EHR and the proposed RSEM, and to functionally provide the user with a CDS system for CRS.

**Figure 4:**
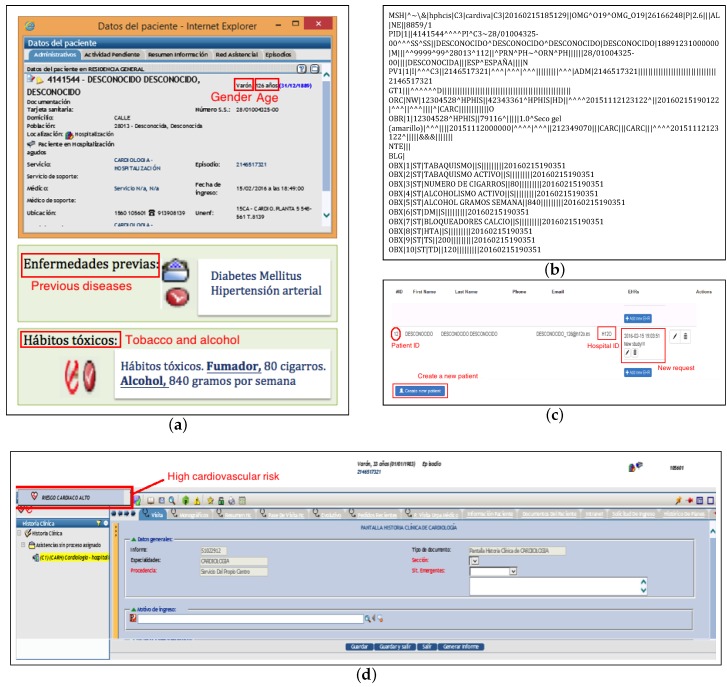
Screenshots to illustrate the workflow between EHR and RSEM: (**a**) demographic and clinical data from a patient recorded in the EHR; (**b**) OMG HL7 message; (**c**) risk stratification request generated in RSEM; (**d**) screenshot of the alert created in the EHR.

The workflow from data of a specific patient in an EHR and the proposed RSEM can be summarized as follows:

1. Demographic and cardiac data from the patient is recorded in the EHR, as shown in Figure 4a.
2. A CRS request is made in the EHR by a clinician. This generates an OMG HL7 message (general clinical order message), which is sent from the EHR to the RSEM following the structure of the patient summary and the HRT and HRV archetypes. This message starts with a header identified by the segment MSH, followed by segments PID (patient identification) and PV1 (patient visit). OBR describes the observation request, and OBX is the observation/result. This segment contains the information related to tobacco or alcohol, among others (see Figure 4b for details).
3. The RSEM receives in real time both the CRS request and the patient summary and HRT and HRV indices, which are saved. Note that RSEM can work with different EHRs from different hospitals, supporting a potential multicentric study. Toward that end, a field indicating the hospital is included (for example H12O, see Figure 4c). Apart from receiving a request from a specific EHR of a hospital, an instance of a new patient can be created manually through the RSEM as shown in Figure 4c.
4. HRT and HRV indices are evaluated, allowing for the classification of patients into the three categories previously described. The evaluation provided by RSEM is sent to the EHR, generating an alert that shows the result of the previous request in Figure 4d. We achieve this by means of two HL7 messages: a PPR message (patient problem message) to send the alert and an ORU message (result message) to finalize the request.

## 4. Discussion

The present work has addressed the proposal and building of a web system, called RSEM, for supporting CRS from ECG monitoring. RSEM provides support to multicentric systems so that the EHR and Holter information can be accessed for CRS using clinical standards.

Much attention has been paid during the last several years to develop and put into practice ontologies and web services to achieve better representations or to build knowledge-based infrastructure for decision-making [36,37,38]. Putting in practice clinical standards to deal with interoperability at different levels of the e-health communication infrastructure for different e-health applications has become a promising line or research [15,39]. Clinical practice and knowledge discovery for clinical decision support was achieved in [40] by building a repository of anonymized information by using CEN/ISO 1360. Authors in [15] describe an open platform to improve the connectivity and reusability of context data to deliver different kinds of health. These systems generate alert messages based on standards related to health care and risk management.

In this work, we go a step further by managing a large amount of clinical data from different entities in an interoperable way, as this can help to extend the knowledge on a specific topic or even to design new CDSSs to assist clinicians in their clinical practice. Though we have not addressed the implementation of an advanced intelligence CDSS from these cases yet, we tested whether the RSEM worked correctly by generating an alert in the EHR under specific conditions in HRT and HRV measurements. The system can be used to give clinical support to health centers with reduced cost and effort, undoubtedly leading to an increase in knowledge of actual HRT and HRV domains, in connection with basic data in the EHR. Another example of highly relevant ECG-derived risk index is the TWA, which consists of changes in a low-voltage scale for waveform characteristics (times, amplitudes, or morphology) of the ST–T complex occurring on a beat-by-beat basis [41,42,43]. This subdomain has not been addressed here, but it is undoubtedly the following one to include in the system.

*Holter-Based Risk Literature.* Many techniques for Holter-based SCD risk stratification have been proposed to date [4] but show limited capabilities mainly due to their poor sensitivity and positive predictive value [17,44,45]. In general, there is no universal index to predict SCD, and the best choice may strongly depend on the pathology under study. For example, in [46], the authors focused on resting the ECG, without considering HRV and HRT. However, they are analyzed in [17], concluding that HRT can be a powerful risk stratification for SCD, whereas further studies are required to conclude the role of HRV. Though in actuality risk is continuous, risk stratification techniques usually dichotomize patients into low- and high-risk groups. The highest-risk subgroups, on which much attention is focused because of the magnitude of the risk of death, actually constitute only a small proportion of the total number of deaths annually. Furthermore, it has been noted that the majority of episodes of SCD actually occur in subjects with low- to intermediate-risk factors and in those without known risk factors. Thus, a comprehensive approach to risk stratification must account for these epidemiological realities. Apart from that, clinical indices arise from many different sources (e.g., ECG, echocardiogram, and blood analysis), which are more or less informative depending on the specific cardiac disease group. Therefore, an approach for studying these cardiac indices should take into account the way they are collected, but special attention must be paid to the origin and causes of SCD.

*Similar Systems and Related Works*. Much attention has been paid over the last several years to developing and putting into practice clinical standards that deal with interoperability at different levels of the communication infrastructure for different e-health applications [39]. In [47], an ontology was developed for yielding interoperability capabilities between clinical research and clinical care domains. A promising line of research that joins clinical practice with knowledge discovery for clinical decision support was achieved in [40] by building a repository of anonymized information using CEN/ISO EN13606. Apart from that, several standard information models based on archetypes for decision support have been proposed in the literature. The authors of [48] discussed and concluded that achieving standardization information models is a complex and time-consuming process that has to take clinicians and technical experts into account. This is one of the reasons of the limited number of real large-scale in-hospital projects (see [49] for a literature review).

In the case of the cardiac domain, several studies can be found. For example, authors in [50] used an open platform and software independent system for diagnosis. They proposed an ontological model for open exchange and representation of ECG data to overcome the differences among the existing formats. The ontology is based on the HL7 medical device communication standard, but no clinical terminologies (such as SNOMED-CT) or clinical standards (such as openEHR) are used. Furthermore, they focus on diagnosis of cardiac abnormalities instead of SCD [51]. The authors of [52] focused on CRS from a complete set of clinical variables to support decision-making. Toward that end, they proposed a web-based system for risk assessment, without considering clinical standards. To the best of our knowledge, no system based on clinical standards for CRS based on ECG monitoring exists.

*Limitations.* Though our focus was not to define new cardiac risk indices, our system allows one to readily include and check new indices that can emerge in the clinical and technical literature. In other words, our target is to contribute to the actual use (or discarding if this is the case) of the existing risk indices. We did not propose here a complete and final system working and connecting several hospitals at this moment, as this will be a developmental project. However, the domain definition in terms compatible with the computational/algorithmic requirements and with the EHR standards turned out to be a complex research work that needed to be addressed separately. For security purposes, we worked with fully anonymized patient data, though VPN-based solutions could also be considered.

## 5. Conclusions

Close cooperation with cardiologists was assessed to define the chosen cardiac risk subdomains. In the long term, the system will allow one to not only include other indices but also work on a large scale of a number of patients. Achieving multicentric connectivity in different hospitals will represent an increase in the scale of collected data. This approach paves the way toward intelligent systems that will take advantage of the current state of knowledge and advances on machine learning models and big data systems so that the CRS in large patient databases can be analyzed.

## Figures and Tables

**Table 1:** Patient summary data description. M: male, F: female.

	General Factors							Diagnosis				Drugs
**Pat.**	**Age**	**Gender**	**Height**	**Weigth**	**BMI**	**Tobac.**	**LVEF**	**Diab.** **mell.**	**Hyper-** **Tension**	**Hyper**- **Cholesterol**	**Myocar.** **Infarct**	β**-Blocker**
1	71	M	90	160	35.16	yes	28	yes	yes	yes	yes	
2	45	M	95	177	30.32	yes	35	no	yes	yes	no	low
3	75	M	81	169	28.36	no	35	no	yes	no	no	
4	60	M	75	175	24.49	yes	35	yes	yes	yes	yes	medium
5	56	M	90	178	28.41	yes	24	no	yes	yes	yes	low
6	60	M	70	170	24.22	no	26	yes	yes	yes	yes	low
7	57	F	94	156	38.63	yes	30	yes	yes	yes	no	
8	59	F	70	150	31.11	yes	29	yes	yes	no	no	low
9	66	F	63	168	22.32	no	35	no	no	no	no	medium
10	67	F	68	155	28.3	no	33	yes	no	no	yes	low
11	51	M	78	180	24.07	yes	32	yes	yes	no	no	
12	50	F	90	160	35.16	yes	19	no	no	no	no	
13	60	M	75	168	26.57	yes	26	yes	yes	no	yes	low
14	70	M	70	165	25.71	no	27	no	yes	no	no	
15	61	F	61	157	24.75	no	21	no	no	no	no	medium
16	78	M	85	167	30.48	no	20	yes	yes	no	no	
17	65	M	102	173	34.08	yes	23	yes	no	no	no	low
18	71	M	78	169	27.31	yes	32	yes	yes	no	yes	low
19	76	M	60	160	23.44	no	26	yes	no	no	no	low
20	63	M	80	161	30.86	no	14	no	yes	yes	no	medium
21	52	M	104	170	35.99	yes	22	no	no	no	no	
22	78	F	87	163	32.74	no	26	yes	yes	yes	no	high
23	57	M	90	170	31.14	yes	37	no	no	no	no
24	50	F	54	167	19.36	yes	22	no	no	no	no

**Table 2:** HRT and HRV indices. Bold numbers are used to identify non-normal values.

	HRT			HRV									
				**Statistical**				**Geometrical**			**Spectral**		
**Pat.**	**TS**	**TO**	**Cat.**	**SDNN**	**SDANN**	**RMSSD**	**pNN50**	**TINN**	**SD1**	**SD2**	**LF**	**HF**	**Cat.**
2	3	**1.77**	1	**70.15**	**64.79**	15.24	**1.17**	242.83	**10.78**	98.61	**113.89**	**22.47**	1
4	7	−0.17	0									
11	**0.41**	−0.37	1	**34.61**	29.37	**10.21**	0.22	142.24	**7.22**	**48.41**	**24.25**	**19.15**	1
12	11.67	−3.41	0									
16	**1.48**	**5.26**	2	**34.74**	**47.28**	48.68	20.13				**62.03**	**91.96**	
18	**1.03**	−0.37	1										
19				**69.50**	**59.18**	**6.02**	0.01	**16.85**	**4.26**	98.19	**4.27**	**3.86**	2
20	**0.81**	**4.70**	0	**13.36**	**5.20**	**13.90**	**1.37**	**28.70**	**9.83**	**16.14**	**21.40**	**43.00**	2
21	**1.75**	−0.22	1	**63.35**	**52.05**	18.94	**2.06**	274.82	**13.39**	88.58	**174.37**	**62.50**	1
23	**0.93**	**0.58**	2									
24	**0.06**	**0.86**	2	**281.31**	**308.13**	33.50	11.36	**119.56**	**23.69**	**397.13**	**24.70**	**16.59**	2
